# Korean Black Goat Extract Exerts Estrogen-like Osteoprotective Effects by Stimulating Osteoblast Differentiation in MC3T3-E1 Cells and Suppressing Osteoclastogenesis in RAW 264.7 Cells

**DOI:** 10.3390/ijms25137247

**Published:** 2024-06-30

**Authors:** Reshmi Akter, Jin Sung Son, Jong Chan Ahn, Md Niaj Morshed, Gyong Jai Lee, Min Jun Kim, Jeong Taek An, Byoung Man Kong, Joong-Hyun Song, Deok Chun Yang, Muhammad Awais, Dong Uk Yang

**Affiliations:** 1 Graduate School of Biotechnology, College of Life Sciences, Kyung Hee University, Yongin-si 17104, Gyeonggi-do, Republic of Korea; reshmiakterbph57@gmail.com (R.A.); songo2@daum.net (J.S.S.); jongchanahn7@gmail.com (J.C.A.); niajmorshed96@khu.ac.kr (M.N.M.); dcyang@khu.ac.kr (D.C.Y.); 2 Hanbangbio Inc., Yongin-si 17104, Gyeonggi-do, Republic of Korea; 3 SD Leo R&D Center, 9–16, Yeonmujang 5-gil, Seongdong-gu, Seoul 04782, Republic of Korea; imcdoctor2@nate.com; 4 SaeromHanbang R&D Center, 76, Cheonseok-gil, Geumcheon-myeon, Naju-si 58216, Jeollanam-do, Republic of Korea; sr8919@naver.com; 5 Happiness Sales Co., Ltd., 403, Water Valley, 8, Dongtanjungsimsangga 1-gil, Hwaseong-si 18455, Gyeonggi-do, Republic of Korea; hp1970@naver.com; 6 Department of Oriental Medicinal Biotechnology, College of Life Sciences, Kyung Hee University, Yongin-si 17104, Republic of Korea; kong2167@naver.com; 7 Department of Veterinary Internal Medicine, College of Veterinary Medicine, Chungnam National University, Daejeon 34134, Republic of Korea; jh.song@cnu.ac.kr

**Keywords:** menopause, osteoporosis, black goat, estrogen, Wnt/β-catenin

## Abstract

Postmenopausal osteoporosis, characterized by an imbalance between osteoclast-mediated bone resorption and osteoblast-driven bone formation, presents substantial health implications. In this study, we investigated the role of black goat extract (BGE), derived from a domesticated native Korean goat, estrogen-like activity, and osteoprotective effects in vitro. BGE’s mineral and fatty acid compositions were analyzed via the ICP-AES method and gas chromatography–mass spectrometry, respectively. In vitro experiments were conducted using MCF-7 breast cancer cells, MC3T3-E1 osteoblasts, and RAW264.7 osteoclasts. BGE exhibits a favorable amount of mineral and fatty acid content. It displayed antimenopausal activity by stimulating MCF-7 cell proliferation and augmenting estrogen-related gene expression (ERα, *ERβ*, and *pS2*). Moreover, BGE positively impacted osteogenesis and mineralization in MC3T3-E1 cells through Wnt/β-catenin pathway modulation, leading to heightened expression of Runt-related transcription factor 2, osteoprotegerin, and collagen type 1. Significantly, BGE effectively suppressed osteoclastogenesis by curtailing osteoclast formation and activity in RAW264.7 cells, concurrently downregulating pivotal signaling molecules, including receptor activator of nuclear factor κ B and tumor necrosis factor receptor-associated factor 6. This study offers a shred of preliminary evidence for the prospective use of BGE as an effective postmenopausal osteoporosis treatment.

## 1. Introduction

Menopause is a natural biological stage in a woman’s life characterized by the cessation of menstruation due to the aging of the ovaries, typically occurring between the ages of 40 and 58 [1]. During this time, the ovaries gradually lose the ability to form ovarian follicles, leading to a decline in estrogen production [2]. It is projected that by 2030, menopausal and postmenopausal women will constitute 1.2 billion individuals worldwide, with approximately 47 million women joining this group annually [3,4]. Among these women, more than 85% will experience menopausal symptoms associated with estrogen deficiency, such as hot flashes, mood swings, and sweating. Additionally, estrogen plays a critical role in suppressing bone resorption and promoting bone formation in a woman’s body. With the decline in estrogen levels, the balance between bone resorption and bone production, maintained by osteoclasts and osteoblasts, respectively, is disrupted. This leads to a decrease in bone mineral density (BMD) and an increased susceptibility to fractures. Consequently, postmenopausal osteoporosis, resulting from the loss of estrogen, is expected to become more prevalent in the coming years, putting 40% of women worldwide at risk for osteoporotic fractures [5].

Hormone replacement therapy has been commonly used to alleviate and treat postmenopausal symptoms. However, the use of hormone therapy has been associated with an elevated risk of conditions such as breast cancer, coronary heart disease, and stroke [6]. Thereby, patients and healthcare professionals have shown increased interest in complementary therapies that utilize natural products, as they are effective with fewer adverse effects.

Since ancient times, goats have been utilized as a source of food by humans. The black goat (*Capra hircus*), a domesticated wild goat belonging to the Bovidae family, is characterized by its small size and dark-colored fur [7]. In the Republic of Korea, the Korean native black goat (KNBG) has traditionally been raised for milk production, although it has also been utilized for meat production [8]. However, due to the distinct flavor and aroma of goat meat, which is less popular among Western consumers, KNBG meat production has been limited [9]. On the other hand, black goat (BG) milk has been utilized in Western countries such as the United States and Europe to produce processed foods like BG milk and cheese [10]. Goat meat, known for its distinctive taste and aroma compared to lamb and mutton, typically possesses a coarse texture and a dark red color [11]. Thereby, in Korea, BG meat has primarily been used for medicinal purposes in the form of extracts rather than as a meat product [12]. Furthermore, BG meat is known to contain higher levels of essential amino acids and fatty acids compared to pork and beef, as well as elevated concentrations of minerals. To meet the increasing demand for BG meat, 542,744 BG hybrids were raised on 14,664 farms in Korea in 2019 [13]. Previous studies have demonstrated that BG meat contains vital fatty acids that play a significant role in the prevention and treatment of various diseases, particularly in women [14,15].

The primary objective of this research was to explore the therapeutic effects of black goat extract (BGE) on its ability to increase bone formation and decrease osteoclastogenesis in patients with insufficient levels of estrogen in the body using an in vitro model.

## 2. Results

### 2.1. Nutritional Content of BGE

The proximate compositions, collagen, fatty acid, and mineral contents of BGE are shown in Table 1. BGE contained 95.55% moisture, 0.2% crude fat, 3.89% crude protein, and 0.36% ash. Similar to this, Choi et al. [16] observed that the proximate composition of BG meat consisted of 74.40% to 76.04% moisture, 19.83% to 20.47% crude protein, 1.64% to 3.56% crude fat, and 1.04% to 1.11% crude ash. Kim et al. [12] reported that the proximate composition of BG meat consisted of 75.00% moisture, 21.60% crude protein, 1.48% crude fat, and 1.04% to 1.41% crude ash (Table 1). Minerals play a vital role in ensuring the healthy growth and development of BG. The mineral composition of meat can vary depending on factors such as feed and the breed of the animal. In the present study, it was found that BGE contained 65.1 mg/100 g of sodium. In the Korea Health Functional Food Association, there are many sections for the identification of chemical profiles. And we selected nine main nutrient analysis parts, which include calorie, carbohydrate, crude protein, crude fat, moisture, ash, sodium, sugars (fructose, glucose, saccharose, maltose, and lactose), saturated fatty acid, trans fatty acid, and cholesterol. Therefore, according to their protocol, only sodium was measured.

**Figure 1:**
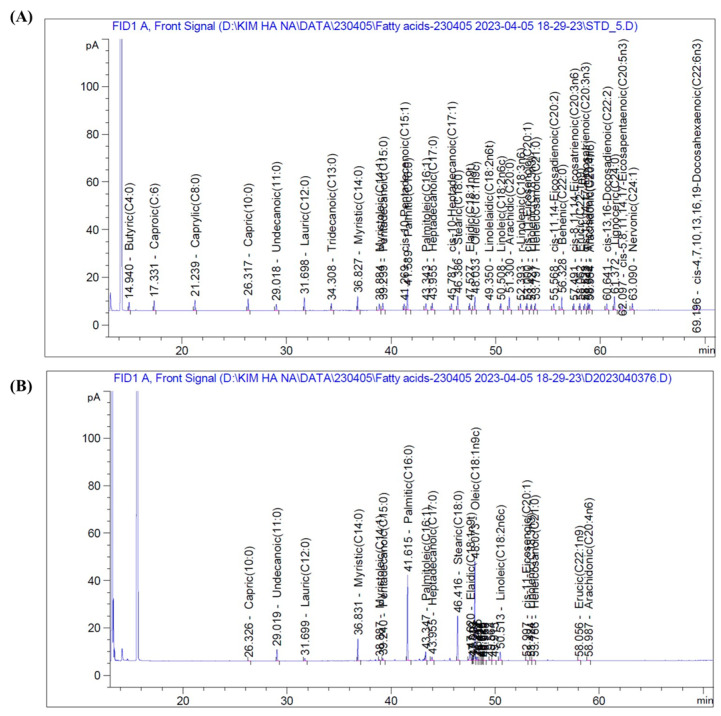
Chemical profile of BGE using a gas chromatography-flame ionization detector (GC-FID): (**A**) fatty acid standards and (**B**) fatty acid detected in BGE.

The composition of fatty acids plays a significant role in shaping the sensory attributes and overall quality of meat. In the context of black goat meat, pivotal fatty acids have been identified, namely, palmitic acid, stearic acid, oleic acid, linoleic acid, and arachidonic acid. Our analysis of black goat meat using Gas Chromatography-Mass Spectrometry (GC-MS) revealed the presence of specific fatty acids: oleic acid with a retention time (RT) of 48.073 min, palmitic acid at RT 41.615 min, stearic acid at RT 46.416 min, myristic acid at RT 36.831 min, elaidic acid at RT 47.620 min, linoleic acid at RT 50.513 min, heptadecanoic acid at RT 43.955 min, and arachidonic acid at RT 58.987 min (Supplementary Table S2A,B) (Figure 1). This analytical approach provides valuable insights into the fatty acid profile of black goat meat, contributing to a deeper understanding of its composition and potential impact on its organoleptic properties and overall quality.

### 2.2. Antioxidant Activity of BGE

**Figure 2:**
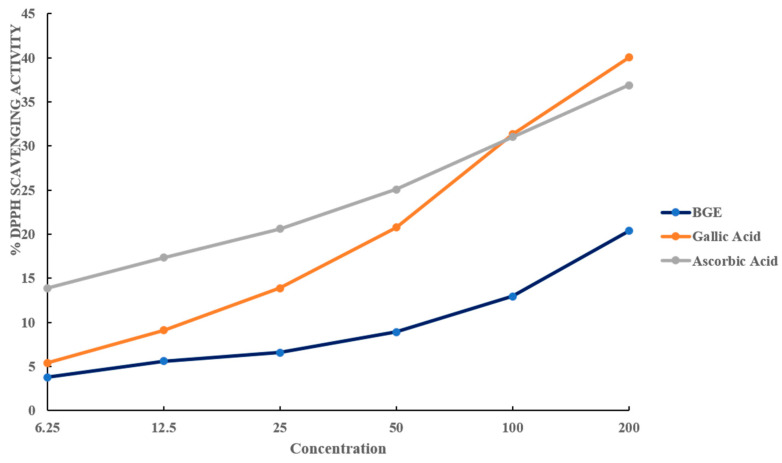
DPPH scavenging activity of black goat compared to gallic acid and ascorbic acid standards.

The DPPH approach is often used to evaluate the antioxidant activity of foods. Diphenyl-picryl-hydrazine, a reliable free radical, was employed to assess the antioxidant activity of our extracts. Supplementary Table S3 displays the antioxidant capacities of BGE. The DPPH results showed that BGE had scavenging capacities of 6191.354 ± 01.011 µg GAE/mg extract and 2855.901 ± 0.01 µg AAE/mg extract. Figure 2 demonstrated that, as compared to conventional ascorbic and gallic acids, BGE’s antioxidant activity increased dose-dependently. Ascorbic acid and gallic acid are widely used as standard antioxidants since they outperform all tested standard compounds in a variety of ways [17].

### 2.3. The Proliferation of Human MCF-7 Cells

**Figure 3:**
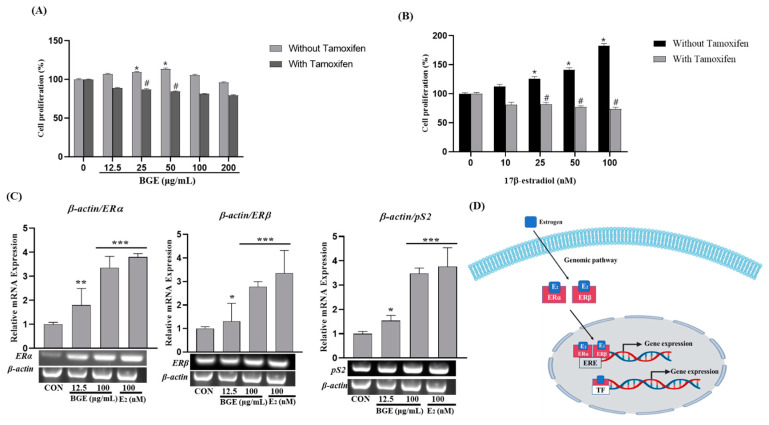
Cell proliferation activity of (**A**) black goat extract (BGE) and (**B**) 17β-estradiol (E_2_) in the absence or presence of tamoxifen, as measured by an E-screen assay in MCF-7 cells. * Significant difference between the cells treated with compounds and the untreated cells. # Significant reduction by co-treatment with tamoxifen compared to treatment with compounds alone. (**C**) Effect of BGE on the transcriptional activation of the *ERα*, *ERβ*, and *pS2* genes in MCF7 cells. MCF7 cells were treated with BGE for 24 h. Subsequently, total RNAs were extracted, and the mRNA expression levels were determined by qRT-PCR analysis and compared with those of *β-actin*. (**D**) The main estrogen receptor signaling pathway. The statistical significance difference was checked using a two-tailed Student’s *t*-test. Data are represented as mean ± SEM. * *p* < 0.05, ** *p* < 0.01, and *** *p* < 0.001 as compared with the control.

We performed a cell proliferation assay using different concentrations of BGE, ranging from 12.5 to 200 μg/mL. According to the findings, all the concentrations of BGE increased cell proliferation except for 200 μg/mL (Figure 3A). In comparison to untreated cells, cell proliferation increased to 113.08 ± 1.39% and 105.5 ± 1.32% after treatment with 50 and 100 g/mL BGE, respectively. However, at 200 µg/mL, the cell survival rate was 95.5 ± 1.25% compared to untreated cells. Nevertheless, the effects were mitigated by tamoxifen, an ER antagonist (Figure 3B). As a result, we have selected a larger range of 100 µg/mL concentrations for the ongoing study. Since E_2_ significantly increased the proliferation of ER-positive MCF-7 cells, it was chosen as a positive control. At 100 nM, E_2_ has shown 182 ± 3.78% proliferation in MCF7 cells compared to untreated cells. These findings demonstrated that BGE might have an E_2_-like action that promoted the growth of ER-positive breast cancer cells.

### 2.4. Effect of BGE on the Gene Expression of ERα and ERβ

Real-Time Quantitative Reverse Transcription PCR was used to assess the expression of ERα and associated pathways to confirm the mechanism of action of BGE on the proliferation of MCF-7 cells (Figure 3C,E). According to our results, the BGE concentrations of 12.5 and 100 μg/mL increased the levels of *ERα*, *ERβ*, and *pS2* gene expressions when compared to untreated cells, whereas the housekeeping gene *β-actin* remained unchanged. Our findings showed that BGE significantly boosted *ERα* and *ERβ* expression in MCF7 cells. In comparison to the expression of E_2_, both genes were considerably expressed.

### 2.5. Effect of BGE on the Alkaline Phosphatase Activity in MC3T3-E1 Cells

**Figure 4:**
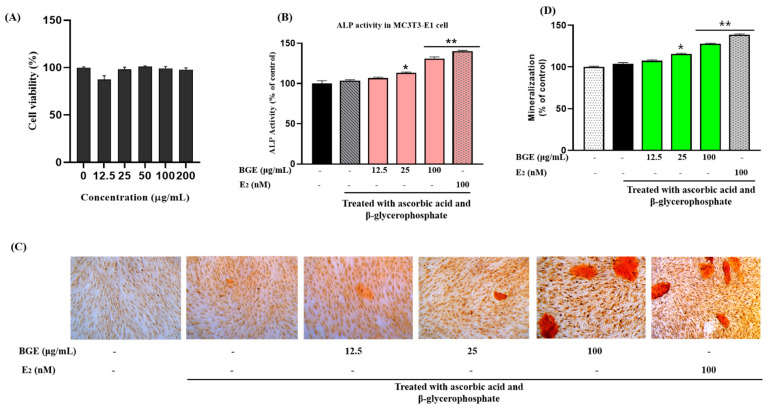
Viability of (**A**) MC3T3-E1 cells incubated with various concentrations of BGE using the MTT assay. (**B**) Effects of BGE on ALP activity in MC3T3-E1 cells. MC3T3-E1 cells were cultured with or without BGE (12.5–100 μg/mL) for 14 d, and the dose-response of BGE extract on ALP activity in cultured cells was assayed. (**C**) Effect of BGE on the mineralization of MC3T3-E1 cells. Extracellular matrix calcium deposits for matrix mineralization were measured using Alizarin Red S dye, which binds to calcium. Treatment with BGE increased extracellular matrix mineralization up to day 28. (**D**) Quantitively analyze the Alizarin Red S dye staining. The statistical significance difference was checked using a two-tailed Student’s *t*-test. Data are represented as mean ± SEM. * *p* < 0.05, ** *p* < 0.01, as compared with the control.

The proliferation of MC3T3-E1 cells was determined using the MTT assay. The results demonstrated that different concentrations of BGE accelerated the proliferation of MC3T3-E1 cells (Figure 4A). Besides, we examined the effect of BGE on alkaline phosphatase (ALP) activity in preosteoblastic MC3T3-E1 cells (Figure 4B). After 14 days of differentiation, the cells treated with BGE exhibited significantly higher ALP activity compared to the control group. To serve as a positive control, E_2_ (100 nM) was utilized. Both BGE at a concentration of 100 μg/mL (30%) and E_2_ (40%) exhibited a substantial enhancement (* *p* < 0.05) in ALP activity when compared to untreated cells. These findings indicate that the BGE heightened ALP activity, potentially contributing to osteoblast development and the mineralization of the extracellular matrix in cases of osteoporosis.

### 2.6. BGE Induces Mineralization and Calcium Deposition in MC3T3-E1 Cells

Furthermore, to evaluate the influence of BGE on the maturation of osteoblasts, we employed ARS staining to quantitatively assess the accumulation of calcium within the extracellular matrix. As illustrated in (Figure 4C), cells subjected to BGE treatment exhibited a modest augmentation in the extent of mineralization. Evident from Alizarin Red S staining, the nodules of calcium deposition displayed a distinct bright orange-red hue. Notably enhanced mineralization was observed up to a concentration of 100 μm of BGE (Figure 4D). When compared to the group that only received ascorbic acid and β-glycerophosphate treatment, the mineralization percentage increased by 3%, 12%, and 25% with BGE treatment at doses of 12.5, 25, and 100 μg/mL, respectively. Moreover, estrogen treatment increased the mineralization area by up to 36%. The experimental timeframe for the mineralization assay spanned 24 days of incubation. Thus, even at lower concentrations of BGE, a substantial enhancement in mineralization and the formation of bone nodules were observable.

### 2.7. BGE Induces Osteogenic Activity via the Wnt Signaling Pathway in MC3T3-E1 Cells

**Figure 5:**
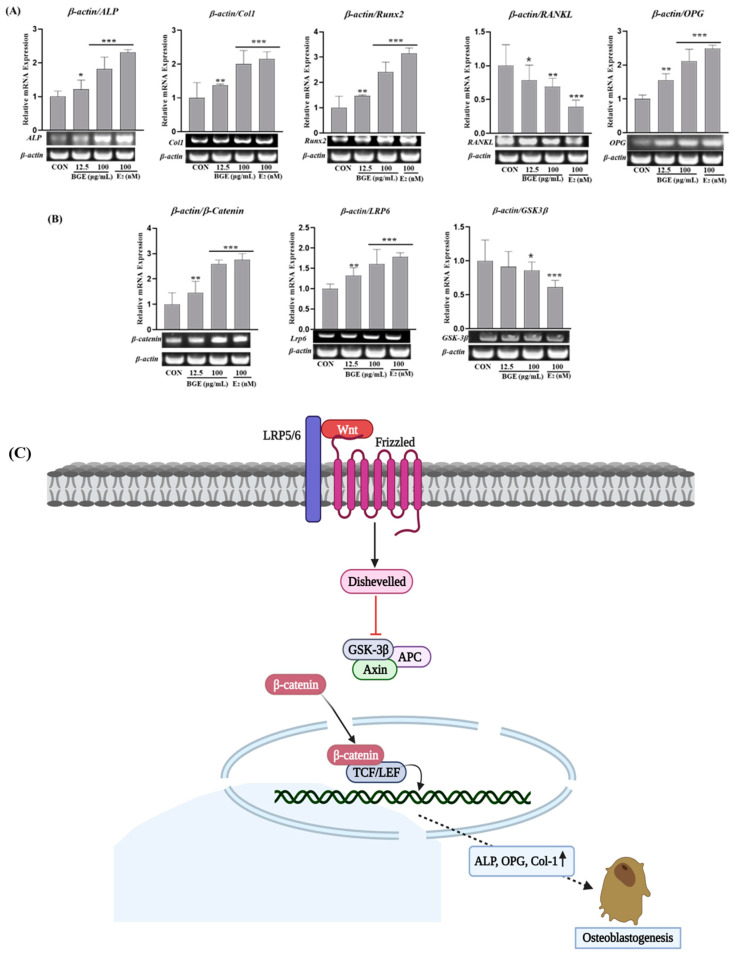
The effects of BGE (**A**) on the mRNA expression levels of Coll-I, RUNX2, ALP, OPG, (**B**) β-catenin, Lrp6, and GSK-3β in MC3T3-E1 cells. mRNA expression levels were assessed by reverse transcription-quantitative polymerase chain reaction analysis; β-actin served as the internal control. (**C**) Wnt signaling is activated by binding to its receptor, which induces the binding of AXIN to phosphorylated lipoprotein receptor-related protein (LRP). The destruction complex is broken, and then β-catenin stabilizes and binds to TCF in the nucleus to regulate the target gene. The data shown are representative of the mean values of three independent experiments, mean ± SEM. * *p* < 0.05, ** *p* < 0.01 and *** *p* < 0.001 vs. untreated control.

Using qRT-PCR, the transcript-level expression of genes encoding osteoblast differentiation marker proteins was examined to better understand how BGE induces growth and differentiation in osteoblastic MC3T3-E1 cells. MC3T3-E1 cells were seeded with differentiation medium (DM) with or without BGE extract into each well of 12-well plates, and they were left to differentiate for a further 14 days. Fresh media, either with or without BGE extract, was used to replace the medium twice a week. To conduct qRT-PCR, total RNA was extracted after 14 days of incubation. In comparison to the control group, there was a significant upregulation (* *p* < 0.05) of Runt-related transcription factor (*Runx2*), *ALP*, collagen type 1 (*Col1a1*), and osteoprotegerin (*OPG*) in the MC3T3-E1 cells treated with BGE (Figure 5A). To clarify the molecular mechanisms behind osteoblast development and activity, which are crucial for bone production, the Wnt/β-catenin signaling pathway was further examined (Figure 5B,C). In osteoblastic MC3T3-E1 cells treated with BGE, mRNA expression of *β-catenin* and *Lrp6* was markedly enhanced (* *p* < 0.05) without showing any discernible change in the level of *GSK-3β* mRNA.

### 2.8. Effect of the BGE on Osteoclastogenesis

**Figure 6:**
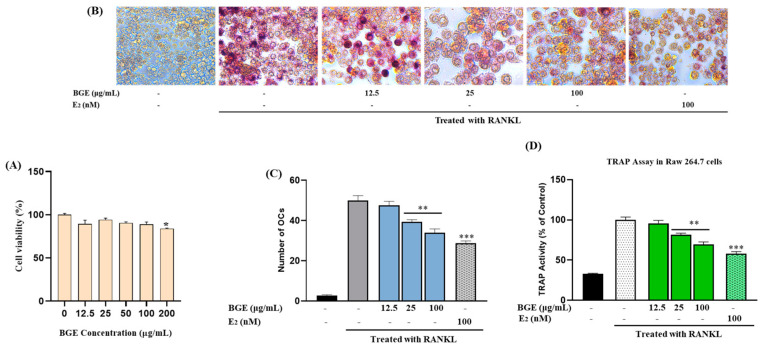
Viability of (**A**) RAW264.7 cells incubated with various concentrations of BGE using the MTT assay. (**B**) RANKL-induced TRAP-positive osteoclast-like cell formation by BGE treatment. Osteoclast differentiation: Multinucleated osteoclasts were visualized at 40× magnification under light microphotography. (**C**) TRAP-positive cells containing more than three nuclei were counted as osteoclasts. (**D**) TRAP activity was measured using the TRAP solution assay. Each value is the mean ± SEM of three independent experiments (* *p* < 0.05, ** *p* < 0.01 and *** *p* < 0.001 vs. RANKL treatment).

Next, we examined the impact of BGE (12.5 to 100 μg/mL) on osteoclast cell survival, and osteoclastogenesis. After 48 h of treatment in RAW264.7 cells, none of the dosages examined resulted in harmful effects (Figure 6A). Thereby, in the presence of RANKL, RAW 264.7 cells produce multinucleated osteoclast-like (OCL) cells that are TRAP-positive (Figure 6B). However, the number of multinucleated cells that were positive for TRAP decreased in BGE-treated (12.5 to 100 μg/mL) RAW 264.7 cells. The tartrate-resistant acid phosphatase (TRAP) assay is the most common method to detect osteoclast populations in vitro. A cell was considered an osteoclast if it was positive for TRAP staining (pink to purple color in Figure 6B) and had more than three nuclei. Furthermore, cells treated with RANKL exhibited an approximate 50% increase in osteoclasts (OCs), while treatment with 100 μg/mL of BGE resulted in a 17% decrease in the number of OCs (Figure 6C). E_2_, on the other hand, led to a reduction of 22% in OCs. Notably, BGE-treated OCL cells in cultures showed morphological variations from control OCL cells, being smaller in size and having fewer nuclei. Similarly, BGE significantly decreased TRAP activity in a dose-dependent manner (Figure 6D).

### 2.9. BGE-Suppressed RANKL-Induced Osteoclast Marker Gene Expression

**Figure 7:**
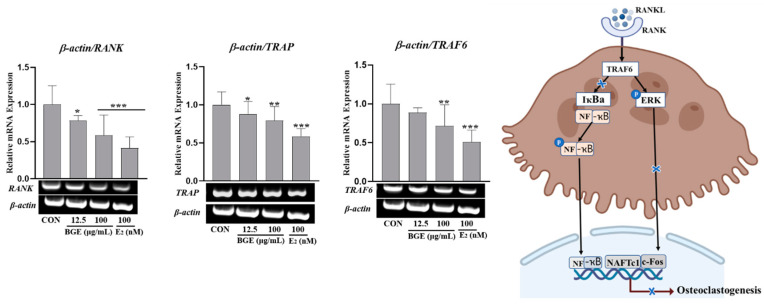
Effects of BGE on the suppression of receptor activator of nuclear factor kappa B ligand (RANKL)-induced mRNA expression level in RAW264.7 cells. Cells were cultured with the indicated concentration of BGE in the presence or absence of RANKL (50 ng/mL). After 7 days, the mRNA expression levels of TRAF6, TRAP, and RANK were determined by qRT-PCR and compared. The results are expressed as the mean ± SEM. (** *p* < 0.01, * *p* < 0.05, *** *p* < 0.001 vs. RANKL-treated cells).

By using qRT-PCR, we initially investigated the potential impact of BGE on the expression of the *RANK* and *TRAF6* genes. The expression of RANK and TRAF6 in RAW 264.7 cells was up-regulated by RANKL stimulation, as shown in Figure 7. However, the enhanced expression of both genes was significantly reduced by BGE treatment. Additionally, the mRNA level of *TRAP* was also significantly higher in RANKL-treated raw 264.7 cells, which was detectably (* *p* < 0.05) diminished in cells treated with BGE. These results suggest that BGE inhibited the gene expression of osteoclast-specific markers that are regulated by NF-κB signaling during osteoclastogenesis.

## 3. Discussion

Black goats have long been recognized for their light carcasses and meat with a distinctive wild flavor. Folk remedies involving extracts from BG meat have been utilized for centuries, benefiting various population groups such as young children, expectant mothers, and senior citizens. The presence of nutritional attributes, coupled with low-fat content, contributes to the potential health advantages of BGE [9]. Furthermore, fatty acids play an important role in reducing the risk of osteoporosis and slowing the rapid rate of postmenopausal bone loss [18]. Previous research has demonstrated a significant correlation between dietary long-chain fatty acids and BMD in postmenopausal women, particularly in the lumbar spine and femoral neck [19,20]. Moreover, antioxidants have been found to be beneficial during the postmenopausal stage of women’s lives. In our study, we evaluated the antioxidant capacity of BGE, as maintaining an adequate antioxidant state and protecting against oxidative stress are vital in the prevention and treatment of postmenopausal osteoporosis [21].

In a previous study, it was demonstrated that fatty acids derived from *Vitex pinnata* L. exhibited estrogenic effects in female Wistar rats [22]. Similarly, BGE contains a significant quantity of fatty acids, which could potentially contribute to its estrogenic activity. Additionally, the substantial nutritional content of the BGE might constitute another influential factor underlying its estrogen-like effects. The E-screen assay, a widely employed cellular proliferation assessment, enables the comprehensive exploration of overall estrogenic activity by observing the augmentation in cell count among estrogen receptor-positive cells, such as the MCF-7 cells. Therefore, as a preliminary step, the E-screen assay was conducted to assess the estrogenic activity of the BGE. Our findings indicated that BGE exhibited a biphasic effect on cell proliferation: it promoted cell proliferation in a dose-dependent manner significantly in the range of 12.5–50 μg/mL, while such promotion was decreased to 100 μg/mL or not observed at higher concentrations. However, the cell proliferative effect was further decreased by co-treatment with the estrogen receptor (ER) antagonist tamoxifen. These results suggest that BGE exhibits physiological activity similar to that of estrogen. Moreover, estrogens and their receptors bind to estrogen response elements (EREs) in the nuclei of breast cancer cells, activating the transcription and expression of proliferation-related target genes [23]. Hence, the proliferation-stimulatory effect of BGE was confirmed through the upregulation of ERα, ERβ, and the estrogen-regulated gene pS2 in the MCF-7 cells.

Furthermore, we evaluated the impact of BGE on factors related to osteoblast and osteoclast differentiation and proliferation. The MC3T3-E1 pre-osteoblastic cell line was employed as an in vitro model for osteogenesis. ALP is a well-known biochemical indicator of osteoblastic activity and is believed to play a role in bone mineralization, although its exact mechanism of action remains unclear. As a result, we investigated the effect of BGE on the ALP activity of osteoblastic MC3T3-E1 cells. ALP activity was elevated by BGE, dose-dependently. Osteoblasts express specific transcription factors such as ALP, Runx2, Col1a1, and OPG during the process of differentiation. Runx2, a transcription factor specific to osteoblasts, plays a significant role in osteogenic differentiation by regulating the expression of genes such as *ALP*, *Col1a1*, and *OCN* [24]. Col1a1, another bone marker, serves as a substrate for calcium deposition and cell adhesion [25]. Compared to the control group, the BGE-treated MC3T3-E1 cells exhibited differential expressions of these osteoblast-related genes during the differentiation process. Additionally, OPG, released by active osteoblasts, functions as a decoy receptor for RANKL, thereby inhibiting RANKL-RANK interaction and limiting osteoclast development and activity during bone remodeling [26]. Previous research suggests that fatty acids primarily confer protective effects on bone health through the enhancement of bone marrow mesenchymal stem cells and osteoblast functions, along with the suppression of osteoclast activities [27]. Moreover, the significant roles of dietary calcium and sodium in upholding bone health among postmenopausal women [28] highlight the potential contribution of sodium present in our BGE as an additional influential factor that may underlie its impact on bone health. Our experiments demonstrated that BGE significantly enhanced *OPG* expression without inducing *RANKL*. These findings suggest that BGE may indirectly control osteoclastogenesis through the release of OPG by active osteoblasts.

The Wnt/β-catenin signaling pathway is widely recognized as a crucial signaling system involved in osteogenic differentiation, bone formation, and the prevention of osteoporosis [29,30]. Wnt ligands can activate the canonical Wnt signaling pathway through the Frizzled receptor and low-density lipoprotein receptor-related protein 5 or 6 (Lrp5/6) receptors at the cell surface [31]. This leads to the stabilization of β-catenin in the cytoplasm, its accumulation in the nucleus, and its interaction with TCF/LEF to promote the expression of downstream target genes. β-catenin, the canonical transcriptional mediator of Wnt signaling, can be released when Wnt ligands bind to their receptors, inhibiting GSK3-β in the cytoplasm. Released β-catenin then translocates into the nucleus, where it regulates the expression of target genes. Disruption of β-catenin stabilization may occur if the GSK3-β complex is not adequately inhibited. This leads us to identify the expression of Lrp6, β-catenin, and GSK-3β expressions following BGE treatment. The findings showed that BGE-boosted *β-catenin* and *Lrp6* gene expression were enhanced by BGE treatment; however, the effect on *GSK-3β* expression was not profound. Our findings were consistent with prior research showing that Wnt/β-catenin signaling accelerated osteoblastic development in vitro [25,32].

BGE also inhibits osteoclast differentiation in Raw 264.7 cells induced by the RANKL. RANKL is a crucial cytokine that promotes osteoclast development, which involves proliferation, fusion, maturation, and resorption stages. Upon binding of RANKL to the RANK receptor on osteoclast precursors, the RANKL/RANK/tumor necrosis factor receptor-associated factor 6 (TRAF6) axis activates downstream signaling pathways, such as the nuclear factor-kappa B (NF-κB) pathway, which, in turn, activates transcription factors required for osteoclast differentiation, activation, and survival [33]. In our study, we found that BGE effectively reduced RANKL-induced osteoclastogenesis without compromising the viability of Raw 264.7 cells. BGE treatment resulted in a significant reduction in the number of multinucleated cells and cells positive for TRAP staining, which is an enzyme associated with bone resorption. Additionally, BGE treatment downregulated the expression of genes involved in mature osteoclast development, namely, *TRAP*, *RANK*, and *TRAF6*. Notably, TRAF6 is a key regulatory factor in RANKL/RANK signaling cascades. Moreover, BGE exhibited a pronounced cytopathic effect and significantly inhibited TRAP activity. These findings collectively suggest that BGE promotes osteoblast differentiation and inhibits osteoclast differentiation in an in vitro setting. Further research is warranted to elucidate the underlying mechanisms and evaluate the efficacy and safety of BGE in clinical settings.

## 4. Materials and Methods

### 4.1. Sample Collection and Preparation

The BG originated from the Republic of Korea, and the raw material supplier was Black Goat Duri Co., Ltd. (Hwasun-gun, Republic of Korea). To prepare the Black Goat Extract (BGE), 1 kg of BG bones from leg and rib meat were combined with 5 L of water and subjected to a 48-h extraction process in a boiling jar, maintaining a meat-to-water ratio of 1:5. Following the extraction, the crude extract underwent a 48-h freeze-drying process to transform it into a powder form. Subsequently, 20 mg of the powder was accurately measured and dissolved in water to achieve the desired concentration for use in subsequent experiments.

### 4.2. Nutritional Value Analysis

All component analysis in this study was analyzed by the Korea Health Functional Food Association Sub. Korea Health Supplement Institute based on the general component analysis method of the Food Code published by the Ministry of Food and Drug Safety in Korea. Moisture content was analyzed by the atmospheric drying method at 105 °C, and ash content was analyzed by the direct ashing method at 550 °C. The crude protein content was analyzed using a Semi-micro Kjeldahl automatic protein analyzer (Kjeltec protein analyzer, Tecator Co., Hoeganaes, Sweden), and the crude fat content was analyzed by solvent extraction. Sugars or saccharides were analyzed using the Bertrand method [19]. Dietary fiber content was analyzed by the enzymatic gravimetric method, and total carbohydrates are expressed as the amount obtained by subtracting the amount of moisture, crude protein, crude fat, and ash from 100 g of sample, and the results of these component analyses are typically expressed as percentages in food analysis.

### 4.3. Mineral Content

One of the major mineral contents (sodium) was measured according to the ICP-AES method in the Food Code (Song and Gil 2002). Briefly, 2 g of individual meat samples underwent ashing at 550 °C. Subsequently, the resulting ashed meat samples were solubilized using 65% nitric acid. The resulting solutions were then carefully transferred into 100 mL volumetric flasks and diluted to their respective volumes using distilled water. The quantification of mineral constituents within the diluted solutions was conducted utilizing an inductively coupled plasma optical emission spectrometer (ICP-MS) and ICP atomic emission spectroscopy (ICP-AES).

### 4.4. Fatty Acid Composition Analysis

The extracted crude fat was treated according to the Fatty Acid Act 2 of the Food Code (Ministry of Food and Drug Safety, 2023) [16], and the test solution was analyzed using a gas chromatography-flame ionization detector (GC-FID) (7890A, Agilent, Santa Clara, CA, USA). Detector flame ionization detector (FID) (Agilent)-8, and the analysis temperature was set at 285 °C. Column SPTM-2560 (Supelco, St. Louis, MO, USA) (100 mx 0.25 mM × 0.20 IM) was used for analysis. The injector was a split mode with a split ratio of 200:1, and the temperature was set at 225 °C. Helium was used as a carrier gas, and the column flow rate was 0.75 mL/min. The temperature of the column was initially maintained at 100 °C for 4 min, then raised to 3 °C per min, maintained at 208 °C for 5 min, then raised to 2 °C per min, and maintained at 244 °C for more than 15 min. In total, 37 components (47885-U, Supelco, St. Louis, MO, USA) were used as the standard material, and each fatty acid methyl ether in the test solution was confirmed by comparing the chromatogram of the standard material with the retention time. The content of saturated fat and trans-fat in the extract was determined by using 37 fatty acid methyl ether standards with carbon coefficients of 4–24.

### 4.5. Cholesterol Analysis

The lipids in the sample are saponified with an ethanol solution of potassium hydroxide at high temperatures. Cholesterol is extracted with hexane, derivatized by etherification with trimethylsilyl (TMS), and then quantified by gas chromatography.

### 4.6. Calculation of Calories

The energy of food and livestock products is calculated by multiplying the content of crude protein, crude fat, and carbohydrates or sugars in 100 g of the sample by the coefficients of protein 4, fat 9, and sugar 4 using Atwater′s coefficient to calculate each energy in kilocalories (Kcal) and expressed as the total. The unit is kilocalorie or kilojoule (KJ), and the conversion from kilocalorie unit to kilojoule unit is according to the following formula: 1 Kcal = 4.184 KJ.

### 4.7. DPPH Scavenging Assay

The 2,2-diphenylpicrylhydrazyl (DPPH) method was employed to assess the free radical scavenging activity of the tested samples, with a slight modification from a previously established method [34]. A solution of DPPH radical was prepared by dissolving 0.2 M DPPH in analytical-grade ethanol. In a 96-well plate, 20 µL of the extract was combined with 180 µL of the DPPH solution and vigorously shaken in the dark for 30 min at a temperature of 25 °C. The same concentrations were employed for gallic acid and ascorbic acid, which served as standards. The absorbance was measured at a wavelength of 517 nm. The percentage inhibition of the samples was calculated using the following formula: (Absorbance of Control-Absorbance of sample/Absorbance of control) * 100.

### 4.8. Cell Culture

Mouse MC3T3-E1 cells, RAW 264.7 macrophage cells, and the ER-positive human breast cancer cell line MCF-7 were procured from the American Type Culture Collection. MCF-7 cells were grown in a complete media consisting of Dulbecco’s Modified Eagle Medium (DMEM with 4500 mg/L of D-glucose, L-glutamine, sodium pyruvate, and sodium bicarbonate) media supplemented with 10% (*v*/*v*) charcoal-stripped fetal bovine serum (FBS) and 1% penicillin-streptomycin (*p*/s) solution. Furthermore, MC3T3-E1 and RAW 264.7 cells were cultured in Minimum Essential Medium (αMEM) supplemented with 10% FBS consisting of 1% penicillin-streptomycin (*p*/s). The cells were cultivated at 37 °C in a humidified 95% air and 5% CO_2_ environment. The DMEM was obtained from Welgene (Daegu, Republic of Korea), FBS and *p*/s were provided by GenDEPOT, and Charcoal-Dextran and 17β-estradiol(E_2_) were obtained from Sigma Aldrich Chemicals (St. Louis, MO, USA).

### 4.9. E-Screen Assay

The previously described protocol was followed for performing the slightly modified E-screen MCF-7 cell proliferation experiment [35]. In brief, confluent MCF-7 cells were seeded into 96-well plates at a density of 1 × 10^4^ cells/well. The cells were then left to adhere for 24 h in an incubator at 37 °C with 5% CO_2_. After adherence, the medium was aspirated, and an estrogen-free medium consisting of 5% charcoal-dextran-stripped human serum and phenol-red-free DMEM (Welgene, Daegu, Republic of Korea) was added. Subsequently, the MCF-7 cells were treated with BGE (12.5–200 µg/mL) and E_2_ (5–100 nM), either with or without tamoxifen (100 nM), for 48 h. After culturing the cells for 48 h, 20 μL of MTT (3-(4,5-dimethyl-2-thiazolyl)-2,5-diphenyl-2-H-tetrazolium bromide) solution was added to the cells, and the cells were incubated for 3 h. Subsequently, the medium was replaced with 100 µL of dimethyl sulfoxide (DMSO). The absorbance was then measured at a wavelength of 570 nm using a microplate reader (BioTek Instruments, Inc., Winooski, VT, USA). The proportion of cell proliferation was expressed relative to the negative control, which was considered to be 100% cell proliferation.

### 4.10. Cell Viability Assay

A colorimetric test based on the absorption of MTT by viable cells was employed to assess cell proliferation and toxicity in MC3T3-E1 cells and RAW 264.7 cells, respectively. The cells were seeded in 96-well plates at a density of 1 × 10^4^ cells per well. After a 24-h incubation period, the cells were exposed to samples at various concentrations and further incubated for 48 h. Following the two-day incubation, 20 µL of MTT solution was added to each well, and the plates were incubated for an additional 3–4 h. Finally, the absorbance was measured at 570 nm after adding 100 µL of DMSO. Relative cell viability (%) was calculated by comparing the absorbance of treated cells to that of control cells that were not subjected to any treatment.

### 4.11. Osteoblast Differentiation and ALP Activity

Prior to initiating osteoblast differentiation, MC3T3-E1 cells were seeded in 12-well plates and allowed to reach a confluence of 80–90%. Following confluence attainment (designated as day 0), osteoblast differentiation was induced using a specialized DM. This medium consisted of a culture medium supplemented with 50 μg/mL L-ascorbic acid and 10 mM β-glycerophosphate. The cells were then incubated for an additional period ranging from 6 to 24 days. Regular media changes were performed every 2 days throughout this period.

For ALP measurement, the cells were treated with DM medium containing varying concentrations of BGE (12.5 to 100 µg/mL), followed by the initiation of osteoblast differentiation, and cultured for an additional 14 days. The medium was changed every 2 days. After 14 days of incubation, the cell monolayer was washed with phosphate-buffered saline (PBS) to measure ALP activity. After that, cells were lysed with 0.1% Triton X-100/PBS, and the supernatants were recovered by centrifuging for five minutes at 12,000 rpm. The collected supernatants were used for an ALP assay using a kit from Sigma Chemical, St. Louis, MO, USA. The total protein concentration was used to normalize the ALP activity using the BCA kit (Sigma Chemical, St. Louis, MO, USA).

### 4.12. Calcium Deposition and Alizarin Red S Staining

On the 24th day of osteoblast differentiation, the cell cultures were carefully washed using PBS and subsequently fixed using 70% ethyl alcohol. To visualize and quantify calcium deposition, the cells were subjected to Alizarin Red S staining (ARS, 40 mM, Sigma-Aldrich) for a duration of 1 h. Following the staining period, the samples were observed using an optical microscope (Eclipse ME600L; Nikon Instruments, Melville, NJ, USA), and representative micrographs were captured. Subsequently, the cells were dissolved in a resolving solution (20% methanol and 10% acetic acid) for 15–18 min after drying to quantify the ARS staining. The resulting solution was subjected to spectrophotometric analysis with an ELISA reader, measuring the absorbance at a wavelength of 570 nm.

### 4.13. Osteoclast Differentiation and Tartrate-Resistant Acid Phosphatase Activity

RAW 264.7 cells were grown in DMEM complete medium at 37 °C in an incubator with 5% CO_2_. As soon as the cells had reached 80% to 90% confluence, they were cultured in 12-well plates (3 × 10^3^ cells/well) with or without 50 ng/mL of receptor activator of nuclear factor-B ligand (RANKL). Then, the cells were differentiated with 50 ng/mL RANKL in the presence or absence of BGE for an additional 3–7 days to develop osteoclasts (OCs). After the OCs had differentiated (at day 7), the cell monolayers were washed with PBS and then lysed with 0.5% Triton X-100. Cell supernatant was collected after centrifuging at 12,000 rpm for 5 min. The supernatant was utilized to measure activity using a TRAP staining kit from Sigma Chemical, St. Louis, MO, USA. Cell counting was used to examine TRAP-positive cells under a light microscope in at least five random fields.

### 4.14. RNA Isolation and Real-Time Reverse Transcription-PCR (qRT-PCR) Analysis

At a density of 5 × 10^4^ cells per well, MCF-7 cells were plated in 12-well plates. The medium was aspirated, and phenol red-free DMEM with or without BGE was added. After 24 h, the cells were washed, and the total RNA was extracted using TriZol LS reagents (Invitrogen, Carlsbad, CA, USA) following the manufacturer’s instructions. Moreover, osteoblasts were differentiated in the presence or absence of BGE, and after 14 days of differentiation, the total RNA was extracted using TriZol LS reagents. Similarly, total RNA was extracted, followed by 7 days of osteoclast differentiation in the presence or absence of BGE. Besides, using a RevertAid First Strand cDNA Synthesis Kit from Thermo Fisher Scientific, Waltham, MA, USA, 20 μL of cDNA was produced from 2.5 ng of RNA by incubating the mixture at 42 °C for 45 min, followed by 70 °C for 5 min, as directed by the manufacturer. The entire work was carried out in a PCR-clean space. Using an Invitrogen SYBR Green qPCR Super Mix UDG kit and an R-Corbett Rotor-Gene Model 6000 (Mortlake, Australia), real-time reverse transcription-PCR (qRT-PCR) was carried out to measure the expression of genes. The relative expression of gene-specific products was assessed and normalized to the corresponding *β-actin* levels using the 2Ct method. All results were confirmed through three independent experiments. The list of primers is provided in Supplementary Table S1.

### 4.15. Statistical Analysis

The data were presented as the mean ± standard error (SE) derived from a minimum of three distinct and independent experimental replicates. Analysis of the data was performed using GraphPad Prism software (GraphPad Software, version 8.0.2, La Jolla, CA, USA). To assess the overall differences between the treated and untreated (control) groups, statistical analyses, including the Student’s *t*-test and two-way analysis of variance, were employed. Statistical significance was determined at a significance level of * *p* < 0.05.

## 5. Conclusions and Limitations

This study presents pioneering evidence that BGE enhances bone formation and inhibits osteoclast differentiation through the regulation of the Wnt/β-catenin pathway and suppression of TRAP, RANK, and TRAF6 expression. Our findings align with previous research indicating that black goat meat combined with medicinal herbs promotes bone formation and reduces bone resorption [36]. Through comprehensive in vitro experiments, we demonstrated BGE’s estrogenic activity, its stimulation of osteoblast differentiation, and its inhibition of osteoclast differentiation. These effects are mediated via modulation of estrogen receptor signaling, activation of the Wnt/β-catenin pathway, and suppression of RANKL-induced osteoclastogenesis. Therefore, BGE holds promise as a novel alternative for osteoporosis prevention and treatment, particularly for improving bone health in aging populations. This study lays the groundwork for future research and development in this area.

Despite its strengths, our study has several limitations. Primarily, our experiments were conducted using secondary cell lines such as MC3T3-E1 and RAW 264.7. While these cell lines are widely accepted for studying osteoblast and osteoclast differentiation, they may not fully represent the in vivo environment. Further investigations are necessary to validate our findings using primary cell lines, such as bone marrow-derived macrophages. Additionally, the small dose used was selected based on in vitro cytotoxicity analysis. The study’s implications also need to be tested in vivo, particularly in ovariectomized models, to better simulate postmenopausal osteoporosis. Finally, clinical trials are crucial to determining the effective dose, efficacy, and safety of BGE in human subjects. Addressing these limitations will provide more comprehensive insights into BGE’s potential as an adjunct therapy or preventive agent for osteoporosis.

## Figures and Tables

**Table 1:** Content of minerals detected in BGE.

Name	Unit	Result
Calorie	Kcal/100 g	17.36
Carbohydrate	%	ND
Crude protein	%	3.89
Crude fat	%	0.2
Moisture	%	95.55
Ash	%	0.36
Sodium	mg/100 g	65.1
Sugars	mg/g	ND
Cholesterol	g/102 g	ND

## Data Availability

All data generated or analyzed during this study are included in this published article.

## Supplementary Materials

The following supporting information can be downloaded at: https://www.mdpi.com/article/10.3390/ijms25137247/s1.

## References

[1] Abdi F., Rahnemaei F.A., Roozbeh N., Pakzad R. (2021). Impact of phytoestrogens on treatment of urogenital menopause symptoms: A systematic review of randomized clinical trials. Eur. J. Obstet. Gynecol. Reprod. Biol..

[2] Grodstein F., Newcomb P.A., Stampfer M.J. (1999). Postmenopausal hormone therapy and the risk of colorectal cancer: A review and meta-analysis. Am. J. Med..

[3] Nowak B., Matuszewska A., Szeląg A., Danielewski M., Dziewiszek W., Nikodem A., Filipiak J., Jędrzejuk D., Bolanowski M., Kucharska A.Z. (2022). Cornelian cherry (*Cornus mas* L.) extract reduces cardiovascular risk and prevents bone loss in ovariectomized Wistar rats. J. Funct. Foods.

[4] Yazdkhasti M., Tourzani Z.M., Roozbeh N., Hasanpour V., Saeieh S.E., Abdi F. (2019). The association between diabetes and age at the onset of menopause: A systematic review protocol. Syst. Rev..

[5] Park J.H., Son Y.J., Lee C.H., Nho C.W., Yoo G. (2020). Circaea mollis Siebold & Zucc. Alleviates postmenopausal osteoporosis in a mouse model via the BMP-2/4/Runx2 pathway. BMC Complement. Med. Ther..

[6] Park E., Lim E., Yeo S., Yong Y., Yang J., Jeong S.-Y. (2020). Anti-Menopausal effects of Cornus officinalis and Ribes fasciculatum extract in vitro and in vivo. Nutrients.

[7] Dong Y., Zhang X., Xie M., Arefnezhad B., Wang Z., Wang W., Feng S., Huang G., Guan R., Shen W. (2015). Reference genome of wild goat (*Capra aegagrus*) and sequencing of goat breeds provide insight into genic basis of goat domestication. BMC Genom..

[8] Kang G., Cho S., Seong P., Park B., Kim S., Kim D., Kim Y., Kang S., Park K. (2013). Effects of high pressure processing on fatty acid composition and volatile compounds in Korean native black goat meat. Meat Sci..

[9] Jeong C.-H., Seo K.-I., Shim K.-H. (2006). Effects of fermented grape feeds on physico-chemical properties of Korean goat meat. J. Korean Soc. Food Sci. Nutr..

[10] Talavera M., Chambers D.H. (2016). Flavor lexicon and characteristics of artisan goat cheese from the United States. J. Sens. Stud..

[11] Kim B.-K., Lee J.-H., Jung D.-J., Cho K.-H., Hwang E.-G., Kim S.-M. (2010). Effects of feeding herb resources powder on meat quality and sensory properties in Korean native black goat. Food Sci. Anim. Resour..

[12] Kim H.-J., Kim H.-J., Jang A. (2019). Nutritional and antioxidative properties of black goat meat cuts. Asian-Australas. J. Anim. Sci..

[13] Kim H.-J., Kim H.-J., Kim K.-W., Lee J., Lee S.-H., Lee S.-S., Choi B.-H., Shin D.-J., Jeon K.-H., Choi J.-Y. (2022). Effect of feeding alfalfa and concentrate on meat quality and bioactive compounds in Korean native black goat loin during storage at 4 °C. Food Sci. Anim. Resour..

[14] Moon S.-H., Kim N.Y., Seong H.-J., Chung S.U., Tang Y., Oh M., Kim E.-K. (2021). Comparative analysis of proximate composition, amino acid and fatty acid content, and antioxidant activities in fresh cuts of Korean native goat (*Capra hircus coreanae*) meat. Korean J. Food Preserv..

[15] Saldeen P., Saldeen T. (2004). Women and omega-3 Fatty acids. Obstet. Gynecol. Surv..

[16] Choi S., Cho Y., Kim M., Chai H., Lee J., Kim Y. (2000). Effect of Castration and Searing of the Musk Gland on Growth Performance and Meat Quality of Korean Native Goats.

[17] Abramovič H., Grobin B., Poklar Ulrih N., Cigić B. (2018). Relevance and standardization of in vitro antioxidant assays: ABTS, DPPH, and Folin–Ciocalteu. J. Chem..

[18] Harris M., Farrell V., Houtkooper L., Going S., Lohman T. (2015). Associations of polyunsaturated fatty acid intake with bone mineral density in postmenopausal women. J. Osteoporos..

[19] Järvinen R., Tuppurainen M., Erkkilä A., Penttinen P., Kärkkäinen M., Salovaara K., Jurvelin J., Kröger H. (2012). Associations of dietary polyunsaturated fatty acids with bone mineral density in elderly women. Eur. J. Clin. Nutr..

[20] Feehan O., Magee P.J., Pourshahidi L.K., Armstrong D.J., Slevin M.M., Allsopp P.J., Conway M.C., Strain J., McSorley E.M. (2023). Associations of long chain polyunsaturated fatty acids with bone mineral density and bone turnover in postmenopausal women. Eur. J. Nutr..

[21] Yang K., Cao F., Xue Y., Tao L., Zhu Y. (2022). Three classes of antioxidant defense systems and the development of postmenopausal osteoporosis. Front. Physiol..

[22] Promprom W., Chatan W., Munglue P. (2020). Effect of *Vitex pinnata* L. leaf extract on estrogenic activity and lipid profile in ovariectomized rats. Pharmacogn. Mag..

[23] Adachi A., Honda T. (2022). Regulatory roles of estrogens in psoriasis. J. Clin. Med..

[24] Jiang H., Zhong J., Li W., Dong J., Xian C.J., Shen Y.-K., Yao L., Wu Q., Wang L. (2020). Gentiopicroside Promotes Osteogenesis and Prevents Bone Loss in Ovariectomized Mice by Modulation of β-catenin-BMP2 Signaling Pathway. https://www.researchsquare.com/article/rs-50702/v1.

[25] Man X., Yang L., Liu S., Yang L., Li M., Fu Q. (2019). Arbutin promotes MC3T3-E1 mouse osteoblast precursor cell proliferation and differentiation via the Wnt/β-catenin signaling pathway. Mol. Med. Rep..

[26] Tsurukai T., Udagawa N., Matsuzaki K., Takahashi N., Suda T. (2000). Roles of macrophage-colony stimulating factor and osteoclast differentiation factor in osteoclastogenesis. J. Bone Miner. Metab..

[27] Bao M., Zhang K., Wei Y., Hua W., Gao Y., Li X., Ye L. (2020). Therapeutic potentials and modulatory mechanisms of fatty acids in bone. Cell Prolif..

[28] Teucher B., Dainty J.R., Spinks C.A., Majsak-Newman G., Berry D.J., Hoogewerff J.A., Foxall R.J., Jakobsen J., Cashman K.D., Flynn A. (2008). Sodium and bone health: Impact of moderately high and low salt intakes on calcium metabolism in postmenopausal women. J. Bone Miner. Res..

[29] Zhang J., Ma Z., Yan K., Wang Y., Yang Y., Wu X. (2019). Matrix Gla protein promotes the bone formation by up-regulating Wnt/β-catenin signaling pathway. Front. Endocrinol..

[30] Yang B., Li S., Chen Z., Feng F., He L., Liu B., He T., Wang X., Chen R., Chen Z. (2020). Amyloid β peptide promotes bone formation by regulating Wnt/β-catenin signaling and the OPG/RANKL/RANK system. FASEB J..

[31] MacDonald B.T., Tamai K., He X. (2009). Wnt/β-catenin signaling: Components, mechanisms, and diseases. Dev. Cell.

[32] Wang X., Tian Y., Liang X., Yin C., Huai Y., Zhao Y., Huang Q., Chu X., Wang W., Qian A. (2022). Bergamottin promotes osteoblast differentiation and bone formation via activating the Wnt/β-catenin signaling pathway. Food Funct..

[33] Yavropoulou M., Yovos J. (2008). Osteoclastogenesis—Current knowledge and future perspectives. J. Musculoskelet. Neuronal. Interact..

[34] Akter R., Kwak G.-Y., Ahn J.C., Mathiyalagan R., Ramadhania Z.M., Yang D.C., Kang S.C. (2021). Protective effect and potential antioxidant role of kakadu plum extracts on alcohol-induced oxidative damage in HepG2 cells. Appl. Sci..

[35] Akter R., Yang D.U., Ahn J.C., Awais M., Nahar J., Ramadhania Z.M., Kim J.Y., Lee G.J., Kwak G.-Y., Lee D.W. (2023). Comparison of in vitro estrogenic activity of Polygoni multiflori Radix and *Cynanchi wilfordii* Radix via the enhancement of ERα/β expression in MCF7 cells. Molecules.

[36] Song H.-N., Leem K.-H., Kwun I.-S. (2015). Effect of water extract and distillate from the mixture of black goat meat and medicinal herb on osteoblast proliferation and osteoclast formation. J. Nutr. Health.

