# Supplementary material for: Korean Black Goat Extract Exerts Estrogen-like Osteoprotective Effects by Stimulating Osteoblast Differentiation in MC3T3-E1 Cells and Suppressing Osteoclastogenesis in RAW 264.7 Cells
Source: Int J Mol Sci. 2024 Jun 30;25(13):7247. doi: 10.3390/ijms25137247 (PMC11241464; doi:10.3390/ijms25137247)
Supplement: Supplementary file 1 [file ijms-25-07247-s001.zip › ijms-3054259-supplementary.pdf]

Table S1. Primers used in this study.

Genes	Forward Primers	Reverse Primers	Reference
ERα	CCGCTCATGATCAAACGCTCTAAG	GCCCTCTACACATTTTCCCTGGTT	
ERβ	TTCCCAGCAATGTCACTAACTT	TTGAGGTTCCGCATACAGA	[1]
pS2	AATGGGCAGCCGTTAGGAAA	GCGCCCAATACGACCAAA	
ALP	CGAGCAGGAACAGAAGTTTGC	TGGCCAAAAGGCAGTGAATAG	
Runx2	ATGGCCGGGAATGATGAGAA	TCTGTCTGTGCCTTCTTGGT	[2]
Coll-I	GATGGATTCCAGTTCGAGTATG	GTTTGGGTTGCTTGTCTG TTTG	
RANKL	GCAGCATCGCTCTGTTCCTGTA	GCATGAGTCAGGTAGTGCTTCTGTG	
OPG	ACAATGAACAAGTGGCTGTGCTG	CGGTTTCTGGGTCATAATGCAAG	[3]
RANK	AAACCTTGGACCAACTGCAC	ACCATCTTCTCCTCCCGAGT	[4]
TRAP	CTGCTGGGCCTACAAATCAT	GGTAGTAAGGGCTGGGGAAG	
TRAF6	AAAGCGAGAGATTCTTTCCCTG	ACTGGGGACAATTCACTAGAGC	[5]
β-Catenin	GATATTGACGGGCAGTATGCAA	AACTGCGTGGATGGGATCTG	
Lrp-6	GGAAGCCTAAGATTGACAGAGCG	GTTGAGTCCGAGCATATTTGAAG	
GSK-3β	ACACCTGCCCTCTTCAACTTTACC	ATTGGTCTGTCCACGGTCTCCA	
β-actin	CCACACCTTCTACAATGAGC	CTCGTAGATGGGCA CAGTGT	

Table S2A. Retention time and peak area correspond to fatty acid standards.

Data File D:\KIM HA NA\DATA\230405\Fatty acids-230405 2023-04-05 18-29-23\STD_5.D
Sample Name: STD_5

Peak #	RetTime [min]	Type	Width [min]	Area [pA*s]	Area %	Name
7	34.308	BB	0.0587	10.52855	1.93970	Tridecanoic(C13:0)
8	36.827	BB	0.0568	21.74068	4.00535	Myristic(C14:0)
9	38.884	VV	0.0598	10.99732	2.02607	Myristoleic(C14:1)
10	39.239	VB	0.0569	11.10143	2.04525	Pentadecanoic(C15:0)
11	41.269	BV	0.0597	11.05910	2.03745	cis-10-Pentadecenoic(C15:1)
12	41.589	VB	0.0574	33.54519	6.18012	Palmitic(C16:0)
13	43.343	VV	0.0617	10.97600	2.02214	Palmitoleic(C16:1)
14	43.955	BB	0.0600	11.32935	2.08724	Heptadecanoic(C17:0)
15	45.787	BB	0.0627	11.32796	2.08698	cis-10-Heptadecanoic(C17:1)
16	46.386	VB	0.0616	22.87190	4.21375	Stearic(C18:0)
17	47.527	BB	0.0624	11.34275	2.08971	Elaidic(C18:1n9t)
18	48.033	VV	0.0640	23.06392	4.24913	Oleic(C18:1n9c)
19	49.350	VV	0.0625	11.31670	2.08491	Linolelaidic(C18:2n6t)
20	50.508	BB	0.0655	11.65952	2.14807	Linoleic(C18:2n6c)
21	51.300	VB	0.0629	23.46703	4.32340	Arachidic(C20:0)
22	52.393	BB	0.0666	11.22833	2.06863	r-Linolenic(C18:3n6)
23	53.000	BB	0.0644	11.38406	2.09732	cis-11-Eicosenoic(C20:1)
24	53.421	BB	0.0666	11.30701	2.08312	Linolenic(C18:3n3)
25	53.797	BV	0.0636	11.90695	2.19365	Heneicosanoic(C21:0)
26	55.568	BB	0.0670	11.56097	2.12991	cis-11,14-Eicosadienoic(C20:2)
27	56.328	BB	0.0650	23.95578	4.41344	Behenic(C22:0)
28	57.491	BV	0.0683	11.59471	2.13613	cis-8,11,14-Eicosatrienoic(C20:3n6)
29	58.062	BV	0.0657	11.70981	2.15733	Erucic(C22:1n9)
30	58.522	VV	0.0684	11.54200	2.12641	cis-11,14,17-Eicosatrienoic(C20:3n3)
31	58.842	VV	0.0636	12.05492	2.22091	Tricosanoic(C23:0)
32	58.984	VB	0.0691	11.61722	2.14027	Arachidonic(C20:4n6)
33	60.641	BV	0.0687	12.10951	2.23097	cis-13,16-Docosadienoic(C22:2)
34	61.372	BB	0.0665	24.15490	4.45012	Lignoceric(C24:0)
35	62.097	VV	0.0713	11.11772	2.04825	cis-5,8,11,14,17-Eicosapentaenoic(C20:5n3)
36	63.090	VB	0.0671	11.94107	2.19994	Nervonic(C24:1)
37	69.196	BB	0.0820	9.88867	1.82182	cis-4,7,10,13,16,19-Docosahexaenoic(C22:6n3)

Table S2B. Retention time and peak area corresponding to fatty acid detected in BGE.

Data File D:\KIM HA NA\DATA\230405\Fatty acids-230405 2023-04-05 18-29-23\D2023040376.D
Sample Name: D2023040376

Peak #	RetTime [min]	Type	Width [min]	Area [pA*s]	Area %	Name
7	34.308		0.0000	0.00000	0.00000	Tridecanoic(C13:0)
8	36.831	VB	0.0562	34.19603	6.09638	Myristic(C14:0)
9	38.887	BV	0.0573	1.79884	0.32069	Myristoleic(C14:1)
10	39.240	VB	0.0571	3.85517	0.68729	Pentadecanoic(C15:0)
11	41.269		0.0000	0.00000	0.00000	cis-10-Pentadecenoic(C15:1)
12	41.615	BB	0.0613	143.17923	25.52561	Palmitic(C16:0)
13	43.347	VV	0.0608	14.40693	2.56843	Palmitoleic(C16:1)
14	43.955	VV	0.0634	6.29207	1.12173	Heptadecanoic(C17:0)
15	45.787		0.0000	0.00000	0.00000	cis-10-Heptadecanoic(C17:1)
16	46.416	BB	0.0672	80.11741	14.28312	Stearic(C18:0)
17	47.620	VV	0.0988	27.76350	4.94960	Elaidic(C18:1n9t)
18	48.073	VV	0.0698	184.51660	32.89513	Oleic(C18:1n9c)
19	49.350		0.0000	0.00000	0.00000	Linolelaidic(C18:2n6t)
20	50.513	VV	0.0679	16.73341	2.98319	Linoleic(C18:2n6c)
21	51.300		0.0000	0.00000	0.00000	Arachidic(C20:0)
22	52.393		0.0000	0.00000	0.00000	r-Linolenic(C18:3n6)
23	52.997	VV	0.0744	1.22184	0.21783	cis-11-Eicosenoic(C20:1)
24	53.424	VV	0.0724	8.84948e-1	0.15777	Linolenic(C18:3n3)
25	53.786	VV	0.0916	1.79438	0.31990	Heneicosanoic(C21:0)
26	55.568		0.0000	0.00000	0.00000	cis-11,14-Eicosadienoic(C20:2)
27	56.328		0.0000	0.00000	0.00000	Behenic(C22:0)
28	57.491		0.0000	0.00000	0.00000	cis-8,11,14-Eicosatrienoic(C20:3n6)
29	58.056	VV	0.0680	6.27240e-1	0.11182	Erucic(C22:1n9)
30	58.522		0.0000	0.00000	0.00000	cis-11,14,17-Eicosatrienoic(C20:3n3)
31	58.842		0.0000	0.00000	0.00000	Tricosanoic(C23:0)
32	58.987	BB	0.0640	9.56183e-1	0.17047	Arachidonic(C20:4n6)
33	60.641		0.0000	0.00000	0.00000	cis-13,16-Docosadienoic(C22:2)
34	61.372		0.0000	0.00000	0.00000	Lignoceric(C24:0)
35	62.097		0.0000	0.00000	0.00000	cis-5,8,11,14,17-Eicosapentaenoic(C20:5n3)
36	63.090		0.0000	0.00000	0.00000	Nervonic(C24:1)
37	69.196		0.0000	0.00000	0.00000	cis-4,7,10,13,16,19-Docosahexaenoic(C22:6n3)

Table S3. DPPH scavenging activity of BGE compared to gallic acid, and ascorbic acid standard.

**Sample**	**DPPH**	
Unit	(µg GAE/ mg extract)	(µg AAE/ mg extract)
BGE	6191.354±01.011	2855.901±0.011

1. Akter, R.; Yang, D. U.; Ahn, J. C.; Awais, M.; Nahar, J.; Ramadhania, Z. M.; Kim, J. Y.; Lee, G. J.; Kwak, G.-Y.; Lee, D. W., Comparison of In Vitro Estrogenic Activity of Polygoni multiflori Radix and Cynanchi wilfordii Radix via the Enhancement of ERα/β Expression in MCF7 Cells. *Molecules* **2023,** 28, (5), 2199.
2. Siddiqi, M. H.; Siddiqi, M. Z.; Ahn, S.; Kim, Y.-J.; Yang, D. C., Ginsenoside Rh1 induces mouse osteoblast growth and differentiation through the bone morphogenetic protein 2/runt-related gene 2 signalling pathway. *Journal of Pharmacy and Pharmacology* **2014,** 66, (12), 1763-1773.
3. Sun, X.; Wei, B.; Peng, Z.; Fu, Q.; Wang, C.; Zhen, J.; Sun, J., Protective effects of Dipsacus asper polysaccharide on osteoporosis in vivo by regulating RANKL/RANK/OPG/VEGF and PI3K/Akt/eNOS pathway. *International journal of biological macromolecules* **2019,** 129, 579-587.
4. Siddiqi, M. H.; Siddiqi, M. Z.; Kang, S.; Noh, H. Y.; Ahn, S.; Simu, S. Y.; Aziz, M. A.; Sathishkumar, N.; Jiménez Pérez, Z. E.; Yang, D. C., Inhibition of osteoclast differentiation by ginsenoside Rg3 in RAW264. 7 cells via RANKL, JNK and p38 MAPK pathways through a modulation of cathepsin K: an in silico and in vitro study. *Phytotherapy Research* **2015,** 29, (9), 1286-1294.
5. Zhao, H.; Lazarenko, O. P.; Chen, J. R., Hippuric acid and 3-(3-hydroxyphenyl) propionic acid inhibit murine osteoclastogenesis through RANKL-RANK independent pathway. *Journal of Cellular Physiology* **2020,** 235, (1), 599-610.
